# Full Validation of Pooled Antibiotic Susceptibility Testing Using CLSI Methods and Performance Criteria in UTI Pathogens

**DOI:** 10.3390/antibiotics14111168

**Published:** 2025-11-20

**Authors:** Richard A. Festa, Frank R. Cockerill, Rick L. Pesano, Emery Haley, Natalie Luke, Mohit Mathur, Xiaofei Chen, Jim Havrilla, Michael Percaccio, Alain Rosas, Jesus Magallon, Shane Erickson, Mandana Ghashghaie, Jade Sinatra, Vicente Gonzalez, David Baunoch

**Affiliations:** 1 Department of Research and Development, Pathnostics, Irvine, CA 92618, USA; rfesta@pathnostics.com (R.A.F.); mpercaccio@pathnostics.com (M.P.); arosas@pathnostics.com (A.R.); jmagallon@pathnostics.com (J.M.); serickson@pathnostics.com (S.E.); mghashghaie@pathnostics.com (M.G.); jsinatra@pathnostics.com (J.S.); vgonzalez@pathnostics.com (V.G.); 2 Partner, Trusted Health Advisors, Orange, CA 92675, USA; frankcockerill@trustedhealthadvisors.us (F.R.C.); rickpesano@trustedhealthadvisors.us (R.L.P.); 3 Department of Clinical Research, Pathnostics, Irvine, CA 92618, USA; ehaley@pathnostics.com (E.H.); nluke@pathnostics.com (N.L.); 4 Department of Medical Affairs, Pathnostics, Irvine, CA 92618, USA; mmathur@pathnostics.com; 5 Department of Data & AI, Pathnostics, Irvine, CA 92618, USA; xchen@pathnostics.com (X.C.); jhavrilla@pathnostics.com (J.H.)

**Keywords:** urinary tract infection, pooled antibiotic susceptibility testing, antibiotic resistance, disk diffusion, broth microdilution

## Abstract

**Background**: Here, we validate a unique and rapid susceptibility assay, Pooled Antibiotic Susceptibility Testing (P-AST), used for complicated, persistent, and recurrent urinary tract infections (UTIs), following Clinical and Laboratory Standards Institute (CLSI) protocols and performance metrics. **Methods**: P-AST™ was validated against the standard disk diffusion method with discrepancy resolution by the broth microdilution reference method. Performance was evaluated for five groups of non-fastidious uropathogenic organisms (Enterobacterales, Enterococci, Staphylococci, *Pseudomonas aeruginosa*, and *Acinetobacter* species) for up to 20 antibiotics, as clinically relevant per group. Fresh (144 monomicrobial and 49 polymicrobial) and frozen (78 monomicrobial and 7 polymicrobial) clinical urine specimens, as well as contrived specimens from pre-characterized frozen “challenge” isolates (52 monomicrobial and 37 polymicrobial), were included. **Results**: P-AST met CLSI target performance criteria of ≥90.0% categorical agreement, <3.0% very major error, <3.0% major error, minor error ≤ 10.0%, or within laboratory standards, and precision > 95.0% across all analysis groups. Across all monomicrobial analyses, there were no very major errors (VMEs), and two major errors (MEs). Across all polymicrobial analyses, there were three VMEs and two MEs. No organism–antibiotic pair analysis had more than a single VME or ME. **Conclusions**: P-AST, a component of the Guidance^®^ UTI assay, demonstrates acceptable performance within the thresholds established by CLSI when compared against standard and reference methods for antibiotic susceptibility testing. Appropriate performance was established in both monomicrobial and polymicrobial specimens for five CLSI-defined groups of uropathogenic bacteria, against up to 20 antibiotics as clinically relevant to each organism group.

## 1. Introduction

Antimicrobial resistance is a significant and growing worldwide cause of death, with 2019 antimicrobial resistance-associated death rates of 51 per 100,000 (41% of infection-related deaths) in the United States and Canada. Urinary tract infections (UTIs) are among the top disease states resulting in these deaths, along with disability-adjusted life-years (DALYs) associated with antimicrobial resistance [1]. Over 600,000 hospitalizations per year in the US are due to complicated UTIs, with over 80% being non-catheter-associated [2]. UTI management also has a high rate of empiric therapy use in the majority of patients, often due to the delayed time to results and high rate of negative and mixed flora/contamination results [3]. This heavy reliance on empiric treatment in the face of increasing antimicrobial resistance leads to treatment failure rates for approximately 17% of UTI patients, and even higher failure rates for patients with recurrent UTI (21%), the elderly (21%), and prior antibiotic use (25%) [4]. One outpatient-based study on UTIs caused by *Enterobacterales* found that 22% of empirically treated patients were prescribed an antibiotic to which the infection was resistant in vitro, and those patients were twice as likely to be hospitalized within 28 days compared to patients empirically prescribed an antibiotic showing susceptibility [5].

Standalone rapid molecular techniques, such as multiplex-polymerase chain reaction (M-PCR), which detect antibiotic resistance (ABR) genes, have been marketed for clinical use as a faster alternative to standard urine culture with antibiotic susceptibility testing (SUC). However, the absence of known ABR genes does not equate to the sample being susceptible to the antibiotic, nor does the presence of an ABR gene necessarily translate to phenotypic resistance [6,7,8]. In addition, a 40% discordance between ABR gene detection and susceptibility phenotypes has been reported previously [9], and commercial panels generally lack ABR gene targets for most first-line UTI antibiotics, particularly nitrofurantoin and fosfomycin [10,11]. Therefore, molecular ABR results alone are insufficient for accurately guiding directed antibiotic selection [12,13].

Better methods for rapidly determining phenotypic antibiotic susceptibility are urgently needed, especially when managing higher-risk and recurrent UTIs [7]. One such method is Pooled Antibiotic Susceptibility Testing (P-AST), a phenotypic fluorescence-based assay that directly measures organism viability through oxidation/reduction activity in the presence of antibiotics. It is a component of the M-PCR/P-AST™ assay (Guidance^®^ UTI, Pathnostics, Irvine, CA, USA), which has previously been associated with significantly improved patient outcomes when used for the management of complicated and recurrent UTIs [3,14]. Compared to the SUC, which usually takes 3–5 days [3,15,16], the M-PCR/P-AST assay provides results within 24 h of specimen receipt [3]. The P-AST portion of the assay is run on all specimens with a non-fastidious bacterial species present at a microbial density above its limit of detection by the M-PCR portion of the assay. Promising overall performance of P-AST when evaluating error rates and agreement was previously reported in both monomicrobial and polymicrobial specimens [17,18]. However, a full Clinical Laboratory Standards Institute (CLSI)-based evaluation for individual organism group–antibiotic performance has not yet been conducted.

Therefore, this study was designed to closely follow CLSI methods and validation metrics [19,20,21,22,23], including the M52 Verification of Commercial Microbial Identification and Antimicrobial Susceptibility Testing Systems, 1st edition guideline document [22] and the Breakpoint Implementation Toolkit (BIT) website [23]. Here, we tested five groups of non-fastidious uropathogenic organisms (Enterobacterales, Enterococci, Staphylococci, *Pseudomonas aeruginosa*, and *Acinetobacter* species, as delineated by the CLSI M100 [19]) against a panel of 20 antibiotics clinically relevant to UTI (the number of relevant antibiotics tested varied by organism group). P-AST results were compared against the standard disk diffusion (DD) method [19,21,22] with discrepancy resolution by the broth microdilution (BMD) reference method [19,20,22] and error resolution by triplicate repeat testing against BMD [22].

Each organism group/antibiotic study was assessed for major errors (MEs), very major errors (VMEs), minor errors (mEs), categorical agreement (CA), and precision, aiming to validate the P-AST component of the Guidance UTI assay, per CLSI methods and metrics.

## 2. Results

### 2.1. Validation of Monomicrobial Specimens

Initial comparison of P-AST to the standard method, DD (total *n* = 274 specimens), in Step I resulted in overall categorical agreement of 2110 out of 2380 comparisons (88.7%). There were a total of 47 very major discrepancies (VMDs), 30 major discrepancies (MDs), and 193 minor discrepancies (mDs). Step II, which involved an initial comparison of P-AST to the BMD reference method for these discrepant specimens, resulted in a total of five VMEs, 14 MEs, and 56 mEs, with the overall categorical agreement increasing to 2351 out of 2426 comparisons (96.9%). These specimens with errors remaining after Step II were processed in Step III with triplicate repeat testing against BMD, resulting in a final total of zero VMEs, two MEs, and 45 mEs, and bringing the overall categorical agreement to 2382 out of 2429 comparisons (98.1%) (Supplemental Tables S1–S5, Table 1, Table 2, Table 3, Table 4 and Table 5).

#### 2.1.1. Monomicrobial Enterobacterales

Full analysis of all 80 monomicrobial specimens resulted only in minor errors, which were < 8.0% for all 18 tested antibiotics clinically relevant to Enterobacterales, and with 14 out of the 22 minor errors exhibiting essential agreement (Table 1). The CA was > 92.0% for all 18 antibiotics (Table 1).

**Table 1:** Monomicrobial Enterobacterales.

Antibiotic	Total *n*	CA %	VME *n* (%)	ME *n* (%)	mE *n* (%)	mE w/EA (% of mE)
Amoxicillin/ Clavulanate	51	98.0	0	0	1 (2.0)	100.0
Ampicillin	37	100.0	0	0	0	N/A
Ampicillin/ Sulbactam	51	92.2	0	0	4 (7.8)	75.0
Cefaclor	51	100.0	0	0	0	N/A
Cefazolin	69	100.0	0	0	0	N/A
Cefepime	51	94.1	0	0	3 (5.9)	0
Ceftazidime	51	96.1	0	0	2 (3.9)	0
Ceftriaxone	51	98.0	0	0	1 (2.0)	100.0
Ciprofloxacin	51	98.0	0	0	1 (2.0)	100.0
Doxycycline	51	98.0	0	0	1 (2.0)	100.0
Fosfomycin	41	95.1	0	0	2 (4.9)	100.0
Gentamicin	50	98.0	0	0	1 (2.0)	100.0
Levofloxacin	51	98.0	0	0	1 (2.0)	0
Meropenem	52	100.0	0	0	0	N/A
Nitrofurantoin	51	94.1	0	0	3 (5.9)	100.0
Piperacillin/ Tazobactam	53	96.2	0	0	2 (3.8)	50.0
Sulfamethoxazole/ Trimethoprim	51	100.0	0	0	0	N/A
Trimethoprim	63	100.0	0	0	0	N/A

CA = categorical agreement; VME = very major error; ME = major error; mE = minor error; mE w/EA = minor error with essential agreement; N/A = not applicable (there were no minor errors).

#### 2.1.2. Monomicrobial Enterococci

Full analysis of all 60 monomicrobial specimens resulted only in minor errors, which were <6.0% for all eight tested antibiotics clinically relevant to Enterococci, and with all seven minor errors exhibiting essential agreement (Table 2). The CA was >94.0% for all eight antibiotics (Table 2).

**Table 2:** Monomicrobial Enterococci.

Antibiotic	Total *n*	CA %	VME *n* (%)	ME *n* (%)	mE *n* (%)	mE w/EA (% of mE)
Ampicillin	52	100.0	0	0	0	N/A
Ciprofloxacin	51	98.0	0	0	1 (2.0)	100.0
Doxycycline	53	94.3	0	0	3 (5.7)	100.0
Fosfomycin	51	98.0	0	0	1 (2.0)	100.0
Levofloxacin	51	100.0	0	0	0	N/A
Linezolid	59	98.3	0	0	1 (1.7)	100.0
Nitrofurantoin	56	98.2	0	0	1 (1.8)	100.0
Vancomycin	56	100.0	0	0	0	N/A

CA = categorical agreement; VME = very major error; ME = major error; mE = minor error; mE w/EA = minor error with essential agreement; N/A = not applicable (there were no minor errors).

#### 2.1.3. Monomicrobial Staphylococci

Full analysis of all 48 monomicrobial specimens resulted in one ME (for sulfamethoxazole/trimethoprim), meeting the CLSI threshold of <3.0% (Table 3). There were no VMEs, and the CA was >97.0% for all nine tested antibiotics clinically relevant to Staphylococci (Table 3). Additionally, the minor error was <3.0% for all nine antibiotics, with one out of the four minor errors exhibiting essential agreement (Table 3).

Through the timepoints from study start to finish, no vancomycin-resistant specimens were identified in fresh clinical specimens, and none were present in the biobank or the CDC challenge organism panel. Only 15 cases of vancomycin-resistant *Staphylococcus aureus* have been reported in the USA to date [24,25], making such specimens unfeasible to collect, and resulting in no VME data for this combination (Table 3).

**Table 3:** Monomicrobial Staphylococci.

Antibiotic	Total *n*	CA %	VME *n* (%)	ME *n* (%)	mE *n* (%)	mE w/EA (% of mE)
Ciprofloxacin	40	97.5	0	0	1 (2.5)	0
Doxycycline	47	97.9	0	0	1 (2.1)	100.0
Gentamicin	46	97.8	0	0	1 (2.2)	0
Levofloxacin	40	97.5	0	0	1 (2.5)	0
Linezolid	46	100.0	0	0	0	N/A
Nitrofurantoin	47	100.0	0	0	0	N/A
Sulfamethoxazole/ Trimethoprim	41	97.6	0	1 (2.9)	0	N/A
Trimethoprim	40	100.0	0	0	0	N/A
Vancomycin	39	100.0	---	0	0	N/A

CA = categorical agreement; VME = very major error; ME = major error; mE = minor error; mE w/EA = minor error with essential agreement; N/A = not applicable (there were no minor errors); --- = no data available.

#### 2.1.4. Monomicrobial *Pseudomonas aeruginosa*

Full analysis of all 47 monomicrobial *Pseudomonas aeruginosa* specimens resulted in one ME (for ceftazidime), meeting the CLSI threshold of <3.0% (Table 4). There were no VMEs, and all seven antibiotics had a CA > 95.0% (Table 4). Additionally, the minor error was <5.0% for all seven tested antibiotics clinically relevant to *Pseudomonas aeruginosa*, with two out of the four minor errors exhibiting essential agreement (Table 4).

**Table 4:** Monomicrobial *Pseudomonas aeruginosa*.

Antibiotic	Total *n*	CA %	VME *n* (%)	ME *n* (%)	mE *n* (%)	mE w/EA (% of mE)
Cefepime	42	100.0	0	0	0	N/A
Ceftazidime	46	95.7	0	1 (2.9)	1 (2.2)	100.0
Ciprofloxacin	42	95.2	0	0	2 (4.8)	50.0
Gentamicin	41	100.0	0	0	0	N/A
Levofloxacin	42	100.0	0	0	0	N/A
Meropenem	42	97.6	0	0	1 (2.4)	0
Piperacillin/ Tazobactam	43	100.0	0	0	0	N/A

CA = categorical agreement; VME = very major error; ME = major error; mE = minor error; mE w/EA = minor error with essential agreement; N/A = not applicable (there were no minor errors).

#### 2.1.5. Monomicrobial *Acinetobacter* Species

Full analysis of all 39 monomicrobial *Acinetobacter* species specimens resulted only in minor errors, which were <10.5% for all ten tested antibiotics clinically relevant to *Acinetobacter* species, with five out of the eight minor errors exhibiting essential agreement (Table 5). The CA was >90.0% for nine of the ten antibiotics. The CA of 89.7% for cefepime is still considered acceptable performance per CLSI guidelines, since 100% of the errors were minor errors, and 75% of the minor errors exhibited essential agreement (Table 5) [21].

**Table 5:** Monomicrobial *Acinetobacter* species.

Antibiotic	Total *n*	CA %	VME *n* (%)	ME *n* (%)	mE *n* (%)	mE w/EA (% of mE)
Ampicillin/ Sulbactam	39	97.4	0	0	1 (2.6)	100.0
Cefepime	39	89.7	0	0	4 (10.3)	75.0
Ceftazidime	39	100.0	0	0	0	N/A
Ceftriaxone	39	94.9	0	0	2 (5.1)	50.0
Ciprofloxacin	39	100.0	0	0	0	N/A
Gentamicin	39	100.0	0	0	0	N/A
Levofloxacin	39	100.0	0	0	0	N/A
Meropenem	39	100.0	0	0	0	N/A
Piperacillin/ Tazobactam	39	97.4	0	0	1 (2.6)	0
Sulfamethoxazole/ Trimethoprim	39	100.0	0	0	0	N/A

CA = categorical agreement; VME = very major error; ME = major error; mE = minor error; mE w/EA = minor error with essential agreement; N/A = not applicable (there were no minor errors).

### 2.2. Validation of Polymicrobial Specimens

Since polymicrobial specimens were assessed for errors for each organism group present, a single specimen could result in an error for multiple organism groups. Initial comparison of P-AST to the standard method, DD (total *n* = 93 specimens), in Step I resulted in overall categorical agreement of 1645 out of 1953 comparisons (84.2%). There were a total of 35 VMDs, 74 MDs, and 199 mDs. Step II, which involved an initial comparison of P-AST to the BMD reference method for discrepant specimens, resulted in a total of nine VMEs, 10 MEs, and 98 mEs, with the overall categorical agreement increasing to 1833 out of 1950 comparisons (94.0%). The specimens resulting in errors were then processed in Step III with triplicate repeat testing against BMD, resulting in a final total of three VMEs (from two specimen-antibiotic test pairs), two MEs (from two specimen-antibiotic test pairs), and 50 mEs (from 37 specimen-antibiotic test pairs), bringing the overall categorical agreement to 1898 out of 1953 comparisons (97.2%) (Supplemental Tables S6–S10, Table 6, Table 7, Table 8, Table 9 and Table 10).

#### 2.2.1. Polymicrobial Enterobacterales

Full analysis of all 70 polymicrobial specimens resulted in two VMEs (one for ampicillin and one for cefazolin), meeting the CLSI threshold of <3.0% for all antibiotics (Table 6). P-AST also resulted in two MEs (one for ampicillin/sulbactam and one for piperacillin/tazobactam), meeting the CLSI threshold of <3.0% for all antibiotics (Table 6). The CA was ≥90.0% for all 18 tested antibiotics clinically relevant to Enterobacterales (Table 6). The minor errors were ≤10.0% for all 18 antibiotics, with 20 out of the 29 minor errors exhibiting essential agreement (Table 6).

**Table 6:** Polymicrobial specimens containing Enterobacterales.

Antibiotic	Total *n*	CA %	VME *n* (%)	ME *n* (%)	mE *n* (%)	mE w/EA (% of mE)
Amoxicillin/ Clavulanate	40	90.0	0	0	4 (10.0)	50.0
Ampicillin	39	97.4	1 (2.9)	0	0	N/A
Ampicillin/ Sulbactam	70	90.0	0	1 (2.9)	6 (8.6)	83.3
Cefaclor	54	98.1	0	0	1 (1.9)	0
Cefazolin	54	98.1	1 (2.0)	0	0	N/A
Cefepime	40	97.5	0	0	1 (2.5)	0
Ceftazidime	49	100.0	0	0	0	N/A
Ceftriaxone	54	100.0	0	0	0	N/A
Ciprofloxacin	40	90.0	0	0	4 (10.0)	75.0
Doxycycline	40	100.0	0	0	0	N/A
Fosfomycin	38	94.7	0	0	2 (5.3)	100.0
Gentamicin	40	100.0	0	0	0	N/A
Levofloxacin	40	92.5	0	0	3 (7.5)	66.7
Meropenem	40	97.5	0	0	1 (2.5)	100.0
Nitrofurantoin	51	90.2	0	0	5 (9.8)	80.0
Piperacillin/ Tazobactam	45	93.3	0	1 (2.9)	2 (4.4)	50.0
Sulfamethoxazole/ Trimethoprim	40	100.0	0	0	0	N/A
Trimethoprim	51	100.0	0	0	0	N/A

CA = categorical agreement; VME = very major error; ME = major error; mE = minor error; mE w/EA = minor error with essential agreement; N/A = not applicable (there were no minor errors).

#### 2.2.2. Polymicrobial Enterococci

Full analysis of all 50 polymicrobial specimens resulted in one VME (for ampicillin), meeting the CLSI threshold of <3.0% (Table 7). There were no MEs (Table 7). The CA was ≥90.0% for all eight tested antibiotics clinically relevant to Enterococci (Table 7). Additionally, the minor error was ≤10.0% for all eight antibiotics, with 10 out of the 12 minor errors exhibiting essential agreement (Table 7).

**Table 7:** Polymicrobial specimens containing Enterococci.

Antibiotic	Total *n*	CA %	VME *n* (%)	ME *n* (%)	mE *n* (%)	mE w/EA (% of mE)
Ampicillin	46	97.8	1 (2.9)	0	0	N/A
Ciprofloxacin	40	90.0	0	0	4 (10.0)	75.0
Doxycycline	37	100.0	0	0	0	N/A
Fosfomycin	37	100.0	0	0	0	N/A
Levofloxacin	50	92.0	0	0	4 (8.0)	75.0
Linezolid	43	100.0	0	0	0	N/A
Nitrofurantoin	41	90.2	0	0	4 (9.8)	100.0
Vancomycin	42	100.0	0	0	0	N/A

CA = categorical agreement; VME = very major error; ME = major error; mE = minor error; mE w/EA = minor error with essential agreement; N/A = not applicable (there were no minor errors).

#### 2.2.3. Polymicrobial Staphylococci

Full analysis of all 31 polymicrobial specimens resulted only in minor errors, which were <7.0% for all nine tested antibiotics clinically relevant to Staphylococci, and with all three minor errors exhibiting essential agreement (Table 8). The CA was >93.0% for all nine antibiotics (Table 8).

**Table 8:** Polymicrobial specimens containing Staphylococci.

Antibiotic	Total *n*	CA %	VME *n* (%)	ME *n* (%)	mE *n* (%)	mE w/EA (% of mE)
Ciprofloxacin	29	100.0	0	0	0	N/A
Doxycycline	31	93.5	0	0	2 (6.5)	100.0
Gentamicin	30	96.7	0	0	1 (3.3)	100.0
Levofloxacin	31	100.0	0	0	0	N/A
Linezolid	30	100.0	0	0	0	N/A
Nitrofurantoin	29	100.0	0	0	0	N/A
Sulfamethoxazole/ Trimethoprim	29	100.0	0	0	0	N/A
Trimethoprim	29	100.0	0	0	0	N/A
Vancomycin	31	100.0	0	0	0	N/A

CA = categorical agreement; VME = very major error; ME = major error; mE = minor error; mE w/EA = minor error with essential agreement; N/A = not applicable (there were no minor errors).

#### 2.2.4. Polymicrobial *Pseudomonas aeruginosa*

After full analysis of all 32 polymicrobial specimens, the only errors were minor errors, which were <7.0% for all seven tested antibiotics clinically relevant to *Pseudomonas aeruginosa*, and with two out of the three minor errors exhibiting essential agreement (Table 9). The CA was >93.0% for all seven antibiotics (Table 9).

**Table 9:** Polymicrobial specimens containing *Pseudomonas aeruginosa*.

Antibiotic	Total *n*	CA %	VME *n* (%)	ME *n* (%)	mE *n* (%)	mE w/EA (% of mE)
Cefepime	30	100.0	0	0	0	N/A
Ceftazidime	31	100.0	0	0	0	N/A
Ciprofloxacin	30	100.0	0	0	0	N/A
Gentamicin	31	96.8	0	0	1 (3.2)	100.0
Levofloxacin	31	100.0	0	0	0	N/A
Meropenem	32	93.8	0	0	2 (6.2)	50.0
Piperacillin/ Tazobactam	30	100.0	0	0	0	N/A

CA = categorical agreement; VME = very major error; ME = major error; mE = minor error; mE w/EA = minor error with essential agreement; N/A = not applicable (there were no minor errors).

#### 2.2.5. Polymicrobial *Acinetobacter* Species

Full analysis of all 32 polymicrobial specimens resulted only in minor errors, which were <4.0% for all 10 tested antibiotics clinically relevant to *Acinetobacter* species, and with all three minor errors exhibiting essential agreement (Table 10). The CA was >96.0% for all 10 antibiotics (Table 10).

**Table 10:** Polymicrobial specimens containing *Acinetobacter* species.

Antibiotic	Total *n*	CA %	VME *n* (%)	ME *n* (%)	mE *n* (%)	mE w/EA (% of mE)
Ampicillin/ Sulbactam	31	100.0	0	0	0	N/A
Cefepime	30	100.0	0	0	0	N/A
Ceftazidime	32	96.9	0	0	1 (3.1)	100.0
Ceftriaxone	31	96.8	0	0	1 (3.2)	100.0
Ciprofloxacin	30	100.0	0	0	0	N/A
Gentamicin	31	96.8	0	0	1 (3.2)	100.0
Levofloxacin	31	100.0	0	0	0	N/A
Meropenem	32	100.0	0	0	0	N/A
Piperacillin/ Tazobactam	30	100.0	0	0	0	N/A
Sulfamethoxazole/ Trimethoprim	30	100.0	0	0	0	N/A

CA = categorical agreement; VME = very major error; ME = major error; mE = minor error; mE w/EA = minor error with essential agreement; N/A = not applicable (there were no minor errors).

### 2.3. Precision (Reproducibility)

Precision was >95.0% for all groups (Supplemental Table S11).

## 3. Discussion

Standard culture is a mainstay of diagnosing infectious diseases, identifying pathogens in infected samples, and determining their antibiotic susceptibility using isolates tested with antibiotic susceptibility testing (AST). Clinicians rely on AST to make informed prescribing decisions that align with good antimicrobial stewardship practices in the face of continuously increasing antimicrobial resistance. This standard method has been in use for over 60 years with minimal change [26,27,28,29,30], and the outcomes for UTI patients in the US today are as follows: > 600,000 hospitalizations/year [2], >1 million emergency room admissions/year [2], > 13,000 deaths/year [31], up to 25% of all women having more than one UTI in their lifetime [32,33], up to 18% of women having multiple recurrent episodes [33], 25% of sepsis cases originating in the urinary tract (urosepsis) [34], high rates of empiric therapy usage [35], and high rates of treatment failure [4]. Patients with recurrent and complicated UTIs, especially the elderly who have elevated risk [36,37,38,39,40], continue to have a significantly reduced quality of life with impacts on their social, personal, and sexual lives [41,42,43,44,45,46,47,48,49,50,51].

Many limitations of AST significantly contribute to this outcome: slow turnaround time often exceeding three days [3,15,16], poor sensitivity for many relevant UTI pathogens (false negative or incomplete results) [28,52,53,54,55,56,57], indeterminate “mixed flora” or “contamination” results in polymicrobial cases [58,59,60], an arbitrary 100,000 colony forming units (CFU)/mL threshold for diagnosis [26,27,28,30,61,62,63], an inability to detect heteroresistance [19,64], and an inability to account for multi-species interactions in polymicrobial infections [65,66,67,68,69,70]. P-AST is a pooled fluorescence-based AST method that measures cell viability through oxidation/reduction activity, with results available within 24 h from the time of specimen receipt, which aims to address these limitations [3,17,18,65].

Due to the longstanding acceptance of isolate AST as the diagnostic standard, it is important that new technologies are thoroughly validated for accuracy. P-AST has been previously validated as a whole assay [17,18], and has also demonstrated significant improvement in complicated and recurrent UTI patient outcomes as part of the Guidance UTI assay [3,14]. Here, we validated the performance of P-AST for each organism group–antibiotic pairing, for both resistant and sensitive phenotypes, with antibiotic breakpoints and relevance determined by CLSI and closely adhering to M52, M100, M02, and M07 protocols and metrics for determining test accuracy [19,20,21,22]. This was performed against a standard AST method, DD, and the reference AST method, BMD, for both monomicrobial and polymicrobial urine specimens across the five uropathogenic organism groups and 20 antibiotics included on the Guidance UTI panel.

Unlike traditional AST methods, including DD and BMD, which rely on an initial culture to isolate colonies for identification and subsequent susceptibility testing, P-AST does not test susceptibility using isolates. Instead, P-AST utilizes a pooled liquid culture, which allows all non-fastidious organisms from the patient’s urine specimen to grow and contribute to the susceptibility profile, more closely representing the infection dynamics occurring in the bladder during UTI treatment. In particular, the pooled culture approach of P-AST is designed to account for heteroresistance, which is possible in monomicrobial or polymicrobial infections [17,18,71], as well as to account for multispecies bacterial interactions that occur in polymicrobial infections [17,18,65]. It also uses a fluorescent marker to directly determine cell death, instead of relying on optical density or manual visual inspection.

With those distinctions in mind, this study was designed to provide an “apples-to-apples” comparison between P-AST and standard isolate AST methods (DD and BMD). The most straightforward comparison is for monomicrobial specimens, where the two methods can be directly compared using CLSI protocols for validating a new assay. Since the purpose of the study was to validate P-AST, rather than to compare the ability of Guidance UTI to detect pathogens that SUC would miss (as reported elsewhere) [72,73,74,75,76,77], all specimens were first tested to ensure that M-PCR and SUC identified the same pathogen(s), and those that differed were excluded. This exclusion favors SUC in terms of assessment of test utility, since multiple reports have shown the importance of those missed identifications [3,14,72,78,79], but they are not relevant to validate the accuracy of P-AST results compared to a standard method when measuring for the same organism(s).

Polymicrobial specimens were also first checked to ensure that Guidance UTI and SUC identified the same organisms for the same reasons as above. This favors SUC by excluding cases where SUC missed identification of some or all pathogens, but allows for an assessment of P-AST against the same organisms tested by the standard method. Generally, all the same methods and metrics from CLSI were used for polymicrobial specimens as in the monomicrobial analysis. However, since CLSI is not written to account for pooled susceptibility, the standard method AST results were assessed by combining isolate susceptibilities into an “overall” susceptibility profile, which categorized a specimen as resistant when any isolate was resistant. This mimics P-AST, which uses the most conservative breakpoint out of all the organisms identified to provide a pooled single result for each antibiotic.

For Enterobacterales, the organism group with the highest prevalence, 85% of specimens were remnant clinical samples, including both fresh, when available (70%), and frozen, as needed (15%). Following standard practice, we then generated contrived specimens from pre-characterized frozen “challenge” organism stocks for the remaining 15%, which covered rare organism–antibiotic resistance mechanisms. For moderately prevalent organism groups, including Enterococci, Staphylococci, and *Pseudomonas aeruginosa*, 74% of specimens were remnant clinical samples, including both fresh, when available (50%), and frozen, as needed (24%). Contrived specimens from stock “challenge” organisms (26%) covered the remainder of rare organism–antibiotic resistance phenotypes. For *Acinetobacter baumannii*, a rare organism with an overall prevalence of 0.4% in our routine clinical samples, it was necessary to use a higher fraction of challenge organisms to have sufficient numbers for the study. For this organism group, fresh clinical samples were used for 7%, frozen clinical samples for 8%, and challenge organisms for 87% of the total specimens.

For both monomicrobial and polymicrobial specimens, P-AST met the following CLSI thresholds: MEs and VMEs < 3%, CA ≥ 90%, mE ≤ 10% or within laboratory standards, and precision > 95% for all five organism groups and 20 antibiotics [22,23]. This involved 104 individual organism group–antibiotic analyses, each having to pass the criteria for VME, ME, mE, precision, and CA%. The vast majority of tests were concordant in Step I, with some requiring a discrepancy analysis in Step II and only a handful requiring triplicate testing to resolve errors in Step III. Possible sources of some of the discrepancies and errors observed in Steps I–II may include well-recognized inherent technical and biological variability factors for AST, such as inoculum effects, operator effects, and variation in microbial strain properties [80,81]. The study analysis was also limited by data points lost to the skip-well phenomenon in BMD [82].

The workflow established for this validation followed the CLSI guidelines for assessing these discrepancies using alternative comparisons [22]. Disk diffusion is a well-established standard method and is referenced in CLSI as such [19,22]. BMD is the established CLSI reference method, which is meant to resolve any discrepancies between the standard assay and the new assay [19,22]. Triplicate testing accounts for any statistical noise or random error [22].

P-AST is a unique method of measuring phenotypic antibiotic susceptibility that aims to address many of the limitations inherent in standard isolate AST techniques, as well as those limitations observed in other rapid AST systems. Several rapid phenotypic AST technologies exist for blood, urine, and other specimen types, such as those detailed in a systemic review by Reszetnik et al. (2024) [83]. These technologies generally have several limitations, such as: non-standardization of the inoculum for direct-from-specimen tests, limitations on the organisms validated (e.g., only Enterobacterales), limited or no organism identification (which restricts MIC breakpoint determinations), and limited sets of antibiotics tested. P-AST uses a standardized inoculum, has a comprehensive panel of UTI pathogens and antibiotics, is performed only following a positive identification by PCR, and sets MIC breakpoints per CLSI guidelines for the identified organisms. There is already a large body of evidence on the Guidance UTI assay, of which P-AST is a component, that demonstrates test accuracy and validity, as well as overall test utility regarding patient outcomes [3,9,14,15,65,72,73,74,75,76,77,78,84]. However, due to its unique nature, and the longstanding tenure of standard isolate AST methods, it is important to thoroughly validate P-AST using the methods described by CLSI documents, the reference guidelines followed by most microbiologists in the US. Prior studies have evaluated P-AST overall [17,18], and this study describes its performance at the organism group–antibiotic level. Here, the technology passes validation across organism groups and antibiotics, in both monomicrobial and polymicrobial specimens, demonstrating validity for its use in recurrent and elevated risk UTIs.

## 4. Materials and Methods

### 4.1. Study Design and Specimen Selection

This study is an evaluation, based on CLSI methods and validation metrics, of the performance of a unique methodology, P-AST, compared to standard and reference isolate AST methods [19,20,21,22,23]. The analysis is primarily based on remnant fresh clinical urine specimens submitted from urology/urogynecology specialist providers in the United States. Fresh clinical specimens (*n* = 144 monomicrobial; *n* = 49 polymicrobial) eligible for the study were included in consecutive order, from 28 May 2025 to 6 October 2025. For rare organisms and phenotypes, previously frozen biobanked remnant clinical urine specimens (*n* = 78 monomicrobial; *n* = 7 polymicrobial) and specimens contrived from stock isolates of previously characterized “challenge” organisms (*n* = 52 monomicrobial; *n* = 37 polymicrobial) were also utilized to supplement the analysis as needed (Table 11). Many of the rare phenotypes requiring the use of biobanked and challenge stock specimens were low-prevalence antibiotic susceptibility phenotypes, such as *Staphylococcus* species resistant to nitrofurantoin, or organisms, such as *Acinetobacter baumannii*, with a low prevalence in either monomicrobial or polymicrobial patient specimens.

Frozen biobanked specimens were selected in reverse consecutive order, such that the most recent specimens were utilized. To ensure that each organism group–antibiotic analysis met CLSI’s minimum “isolate” number requirements, polymicrobial specimens were counted based on each organism present, rather than counted as a single specimen identity. Thus, polymicrobial specimens were counted toward the “*n*” of each organism group present in the specimen (e.g., a single specimen with both *E. coli* and *E. faecalis* present would be counted toward the “*n*” of Enterobacterales as well as toward the “*n*” of Enterococci). If two organisms in the same specimen represented the same organism group (e.g., *E. coli* and *K. pneumoniae)*, that specimen was only counted once toward the group “*n*”. Fastidious organisms present in polymicrobial specimens were not considered, as they were previously shown not to impact P-AST results [17]. Stock “challenge” organisms were sourced from the United States Centers for Disease Control and Prevention (CDC). In some cases of rare organisms or susceptibility phenotypes for polymicrobial analysis, two or more challenge organism isolates were pooled together to generate a contrived polymicrobial challenge specimen.

The information from fresh and frozen clinical specimens was used in such a manner that the identity of the subject could not be readily ascertained directly or through identifiers linked to the subjects, the subject was not contacted, and the investigator did not re-identify subjects. Therefore, the Western Institutional Review Board deemed the use of the data to be exempt under 45 CFR § 46.104(d)(4).

For this analysis, the inclusion criteria for remnant fresh clinical urine specimens were as follows: (1) submitted with ICD-10-CM codes consistent with a clinical diagnosis of suspected UTI [3]; (2) were a minimum volume of 2 mL urine, collected in boric acid stabilizer (gray-top vacutainers, BD, Franklin Lakes, NJ, USA), kept refrigerated (4 °C) after receipt by the laboratory, and within stability (within 7 days of specimen collection); (3) the same non-fastidious, bacterial species identified by both M-PCR and SUC in parallel. Ensuring the same organism(s) are identified by M-PCR and SUC allows for an apples-to-apples comparison of phenotypic antibiotic susceptibility methods (P-AST vs. DD/BMD isolate AST).

Frozen clinical specimens with appropriate UTI-related ICD-10-CM codes were included to supplement the fresh clinical specimens when (1) the total “*n*” for a particular organism group was less than 30, or (2) when representative specimens with specific, rarer phenotypic populations (susceptible or resistant to a particular antibiotic) were needed. For these biobanked frozen specimens, the organism identity and P-AST results were previously recorded. Specimens with the necessary organism identity and/or phenotype were selected in reverse-consecutive order, with the most recently frozen specimens prioritized. Frozen specimens were thawed from 50% glycerol stocks stored at −80 °C, streaked onto 5% sheep blood agar (Hardy Diagnostics, Santa Maria, CA, USA) to recover isolates, re-constituted in filter-sterilized human urine as the medium, and then subjected to the same M-PCR, P-AST, SUC, DD, and BMD protocols as the fresh clinical specimens.

Finally, frozen stock “challenge” organisms were included to supplement the fresh and frozen clinical specimens when (1) the total “*n*” for a particular organism group was less than 30, or (2) when representative specimens with specific, rarer phenotypic populations (susceptible or resistant to a particular antibiotic) were needed. After selection for the necessary phenotype, these stock isolates were streaked onto 5% sheep blood agar (Hardy Diagnostics, Santa Maria, CA, USA) and passaged a second time, as per CDC instructions, to recover isolates. After isolates were recovered, they were reconstituted in filter-sterilized human urine and processed for AST the same way as the fresh clinical urine specimens.

For each organism group–antibiotic analysis, data was collected to have a minimum of 30 specimens, and to include specimens with both resistant and susceptible results by the standard method. Once each individual organism group–antibiotic analysis was completed, that group was closed to analysis, and no additional data was added. Therefore, for polymicrobial specimens, the data for common phenotypes (e.g., Enterobacterales susceptible to Piperacillin/Tazobactam) was completed before rare phenotypes (e.g., Staphylococci resistant to Vancomycin), and further specimens would only contribute data to the organism–antibiotic analyses that were still collecting data.

### 4.2. Bacterial Identification with Multiplex-Polymerase Chain Reaction

The M-PCR assay was performed as previously described [3]. Briefly, microbial DNA was extracted from the urine samples, mixed with a universal PCR master mix (Thermo Fisher, Carlsbad, CA, USA), amplified using TaqMan technology, and spotted in duplicate on OpenArray chips (Thermo Fisher Scientific, Wilmington, NC, USA) containing the probes and primers used to detect the 13 non-fastidious bacterial species and two non-fastidious bacterial groups included in the analysis:

Enterobacterales [*Citrobacter freundii* (*C. freundii*), *Citrobacter koseri* (*C. koseri*), *Escherichia coli* (*E. coli*), *Klebsiella oxytoca* (*K. oxytoca*), *Klebsiella pneumoniae* (*K. pneumoniae*), *Morganella morganii* (*M. morganii*), *Proteus mirabilis* (*P. mirabilis*), *Providencia stuartii* (*P. stuartii*), *Serratia marcescens* (*S. marcescens*), and the Enterobacter group including *Klebsiella aerogenes* (*K. aerogenes*) (formally known as *Enterobacter aerogenes*) and *Enterobacter cloacae* (*E. cloacae*)]; Enterococci [*Enterococcus faecalis* (*E. faecalis*)]; *Pseudomonas aeruginosa* (*P. aeruginosa*); *Acinetobacter* species [*Acinetobacter baumannii* (*A. baumannii*)]*;* and Staphylococci [(*Staphylococcus aureus* (*S. aureus*) and the Coagulase-negative *Staphylococcus* group (CoNS), including *Staphylococcus epidermidis*, *Staphylococcus haemolyticus*, *Staphylococcus lugdunesis*, and *Staphylococcus saprophyticus*)].

### 4.3. Bacterial Identification with Standard Urine Culture

Bacterial identification by SUC was performed in accordance with the American Society for Microbiology Clinical Microbiology Procedures Handbook, as previously described [3,75,85].

### 4.4. Pooled Antibiotic Susceptibility Testing

Each specimen was tested for susceptibility to the relevant antibiotics for each organism group, as determined by CLSI M100 35th edition [19], out of a total of 20 P-AST panel antibiotics. The 20 UTI-relevant antibiotics on the test panel are the following: amoxicillin/clavulanate, ampicillin, ampicillin/sulbactam, cefaclor, cefazolin, cefepime, ceftazidime, ceftriaxone, ciprofloxacin, doxycycline, fosfomycin, gentamicin, levofloxacin, linezolid, meropenem, nitrofurantoin, piperacillin/tazobactam, sulfamethoxazole/trimethoprim, trimethoprim, and vancomycin.

The P-AST component of the Guidance UTI diagnostic assay utilizes a unique pooled strategy to determine the susceptibility of the entire microbial pellet from a clinical urine specimen. P-AST utilizes resazurin, a fluorescent indicator of metabolic activity, to rapidly detect antibiotic resistance. The assay was performed as described previously [3,17,73]. Briefly, a pre-culture of a microbial pellet was diluted to a final concentration of around 500,000 cells/mL in Mueller–Hinton Growth media and inoculated into a 96-well plate pre-loaded with antibiotics at dilutions spanning all relevant MICs. The test plate and a control plate were incubated for 12–16 h at 35 °C, and the fluorescent intensity of the samples was measured on an Infinite M Nano+ Microplate Reader (TECAN, Männedorf, Switzerland) with a pre-determined fluorescent threshold indicating growth. The unique use of resazurin as a fluorescent indicator of metabolic activity means that growth indicative of antibiotic resistance is highly sensitive and can be detected earlier in the culture incubation period, compared to optical density, which requires that many cell divisions occur to generate enough turbidity for detection, and thus requires a longer incubation time. The P-AST incubation time has specifically been optimized to account for slower-growing organisms, including the *Pseudomonas* and *Enterococcus* species included in this study.

MICs and their associated categorical susceptibility interpretations were determined according to the CLSI M100 Performance Standards for Antimicrobial Susceptibility Testing, 35th edition supplement document [19]. Since the CLSI M100 does not include susceptibility interpretation breakpoints for the combination of *P. aeruginosa* and gentamicin, the breakpoints listed on the US Food and Drug Administration (FDA) Susceptibility Test Interpretation Criteria (STIC) website were utilized [86]. For monomicrobial samples, the P-AST result using these breakpoint cutoffs was directly compared to standard method results.

As previously described, the P-AST categorical susceptibility interpretations for polymicrobial specimens are based on the most restrictive minimum inhibitory concentration (MIC) among all detected non-fastidious organisms [17]. For example, in a monomicrobial specimen, a ciprofloxacin MIC of 1 µg/mL would be interpreted as “resistant” for *E. coli* and “susceptible” for *E. faecalis* specimens, per the breakpoints in CLSI M100 for each species. In a polymicrobial specimen with both *E. coli* and *E. faecalis*, if the P-AST pooled MIC for ciprofloxacin was 1 µg/mL, the test would result that antibiotic as “resistant,” based on the more stringent breakpoints for *E. coli*.

### 4.5. Disk Diffusion Antibiotic Susceptibility Testing

DD was performed on isolates from the SUC plates according to the CLSI M02 Performance Standards for Antimicrobial Disk Susceptibility Test, 14th edition standards document [21], as previously described [18]. Clearance zones were measured manually by ruler by the same research associate and interpreted according to the CLSI M100, 35th edition [19]. Since the CLSI M100 does not include susceptibility interpretation breakpoints for the combination of *P. aeruginosa* and gentamicin, the breakpoints listed on the FDA STIC website were utilized [86].

### 4.6. Broth Microdilution Antibiotic Susceptibility Testing

BMD was performed on isolates from the SUC plates according to the CLSI M07 Methods for Dilution Antimicrobial Susceptibility Tests for Bacteria That Grow Aerobically, 12th edition standards document [20], as previously described [18]. MICs and associated categorical susceptibility interpretations were determined according to the CLSI M100, 35th edition [19]. Since the CLSI M100 does not include susceptibility interpretation breakpoints for the combination of *P. aeruginosa* and gentamicin, the breakpoints listed on the FDA STIC website were utilized [86].

### 4.7. Considerations for Comparing Results for Polymicrobial Specimens

For polymicrobial specimens, to ensure that this study provided equivalent comparisons between the isolate AST and P-AST methods, all isolate AST results were combined into an “overall” susceptibility profile, according to the following rules: (1) If all of the organisms identified were determined to be sensitive to an antibiotic, the overall result was designated to be “sensitive”; (2) If any one organism was determined to be resistant to an antibiotic, the overall result was determined to be “resistant.” This “overall” susceptibility profile was compared to the P-AST result as previously described [17].

### 4.8. Workflow

**Figure 1:**
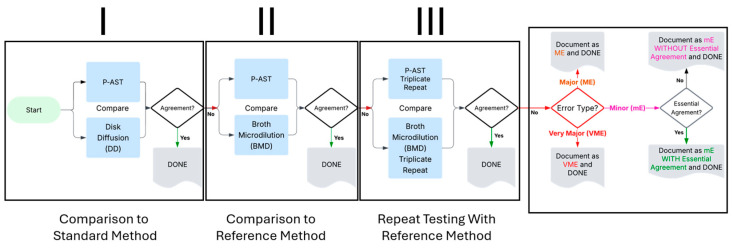
Overview of study workflow. P-AST = Pooled Antibiotic Susceptibility Testing.

The workflow (see Appendix A for more detail) begins with a comparison of the P-AST method to a standard AST comparator method, DD, as described in the CLSI M52 document (Figure 1, Step I) [22]. For specimens resulting in discrepancies, Step II attempts discrepancy resolution, following M52 guidance, by comparing P-AST to BMD, the only reference AST comparator method recognized by CLSI as of the 35th edition of the M100 document [19]. To attempt resolution of any remaining errors, P-AST and BMD testing were repeated in triplicate, as per the M52 document guidelines (Figure 1, Step III).

### 4.9. Precision (Reproducibility)

The precision (reproducibility) of P-AST was validated following the CLSI M52 methods and metrics [22], to assess category equivalency among triplicate P-AST results. Seven representative monomicrobial specimens per organism–antibiotic analysis pair were included in the precision calculations.

### 4.10. Statistical Analysis

Metrics of AST verification were calculated according to CLSI M52 standards from the Verification of Commercial Microbial Identification and Antimicrobial Susceptibility Testing Systems, 1st edition guideline document [22], and the BIT website [23]. In this analysis, in accordance with the M52 [22], differences between P-AST and the DD standard comparator method results are described as discrepancies (VMDs, MDs, and mDs), while differences between P-AST and the BMD reference comparator method are described as errors (VMEs, MEs, and mEs). For simplicity, only the error terminology is used in the table of formulas (Table 12).

The precision (reproducibility) of P-AST was calculated using the CLSI BIT tool formulas (Supplemental Table S11) [23].

## 5. Conclusions

P-AST, a unique component of the Guidance^®^ UTI assay, which rapidly measures the pooled susceptibility of cultivable bacterial organisms from a clinical urine specimen, demonstrates acceptable performance within the thresholds established by CLSI, when compared against standard and reference methods for AST. Appropriate performance was established, using a combination of fresh and frozen clinical specimens plus challenge isolates, in both monomicrobial and polymicrobial specimens, for five CLSI-defined groups of non-fastidious uropathogenic bacteria (Enterobacterales, Enterococci, Staphylococci, *P. aeruginosa*, and *Acinetobacter* species) for up to 20 antibiotics, as clinically relevant to each organism group.

## Figures and Tables

**Table 11:** Specimen source summary.

Microorganism Analysis Group	Remnant Clinical Specimens		Challenge Stocks *n* (%)	Total Specimens *n*
	Fresh *n* (%)	Frozen (Biobanked) *n* (%)		
Monomicrobial				
Enterobacterales	60 (75)	17 (21)	3 (4)	80
Enterococci	35 (58)	25 (42)	0	60
Staphylococci	24 (50)	14 (29)	10 (21)	48
*P. aeruginosa*	22 (47)	18 (38)	7 (15)	47
*Acinetobacter* spp.	3 (8)	4 (10)	32 (82)	39
Polymicrobial				
Enterobacterales	44 (63)	6 (9)	20 (29)	70
Enterococci	38 (76)	6 (12)	6 (12)	50
Staphylococci	7 (23)	2 (6)	22 (71)	31
*P. aeruginosa*	8 (25)	0	24 (75)	32
*Acinetobacter* spp.	2 (6)	0	30 (94)	32

**Table 12:** P-AST performance metric calculation formulas.

P-AST Performance Metric	Calculation Formula
Categorical Agreement (%)	N_CA_/NT × 100
Minor Errors (%)	N_mE_/NT × 100
Minor Errors with Essential Agreement (%)	N_mEEA_/N_ME_ × 100
Very Major Errors (%)	N_VME_/N_RefR_ × 100
Major Errors (%)	N_ME_/N_RefS_ × 100

Key: comparator method = DD standard method, initially; BMD reference standard method is the comparator for cases with initial P-AST/DD discrepant results; MIC = minimum inhibitory concentration; NT = number of organism–antibiotic pair susceptibility test results performed; N_CA_ = number of P-AST results with the same categorical interpretation [“susceptible (S)” or “intermediate (I)” or “resistant (R)”] as the comparator method; N_mE_ = number of minor errors when A) the P-AST categorical result is “intermediate (I)” and the comparator result is either “susceptible (S)” or “resistant, or B) the P-AST categorical result is either “susceptible (S)” or “resistant (R)” and the comparator result is “intermediate (I)”; N_mEEA_ = number of minor errors with essential agreement; essential agreement = when P-AST MIC results are within ± one two-fold dilution of the BMD reference standard MIC results; N_VME_ = number of false-susceptible results by P-AST versus the comparator; N_ME_ = number of false-resistant results by P-AST versus the comparator; N_RefR_ = number of resistant results by the comparator; N_RefS_ = number of susceptible results by the comparator.

## Data Availability

The original data presented in this study are openly available in FigShare at https://doi.org/10.6084/m9.figshare.30311740.

## Supplementary Materials

The following supporting information can be downloaded at https://www.mdpi.com/article/10.3390/antibiotics14111168/s1, Table S1: Monomicrobial Enterobacterales Numbers of Discrepancies and Errors; Table S2: Monomicrobial *Pseudomonas aeruginosa* Numbers of Discrepancies and Errors; Table S3: Monomicrobial Staphylococci Numbers of Discrepancies and Errors; Table S4: Monomicrobial Enterococci Numbers of Discrepancies and Errors; Table S5: Monomicrobial *Acinetobacter* Species Numbers of Discrepancies and Errors; Table S6: Polymicrobial Enterobacterales Numbers of Discrepancies and Errors; Table S7: Polymicrobial *Pseudomonas aeruginosa* Numbers of Discrepancies and Errors; Table S8: Polymicrobial Staphylococci Numbers of Discrepancies and Errors; Table S9: Polymicrobial Enterococci Numbers of Discrepancies and Errors; Table S10: Polymicrobial *Acinetobacter* Species Numbers of Discrepancies and Errors; Table S11: Precision.

## Abbreviations

The following abbreviations are used in this manuscript:

ABR	Antibiotic Resistance
AST	Antibiotic Susceptibility Test(ing)
BIT	Breakpoint Implementation Toolkit
BMD	Broth Microdilution
CA	Categorical Agreement
CDC	United States Centers for Disease Control
CFU	Colony Forming Unit
CLSI	Clinical and Laboratory Standards Institute
DALY	Disability Adjusted Life Years
DD	Disk Diffusion
EA	Essential Agreement
FDA	United States Food and Drug Administration
MD	Major Discrepancy
mD	Minor Discrepancy
ME	Major Error
mE	Minor Error
MIC	Minimum Inhibitory Concentration
M-PCR	Multiplex-Polymerase Chain Reaction
N/A	Not Applicable
P-AST	Pooled Antibiotic Susceptibility Test(ing)
STIC	Susceptibility Test Interpretive Criteria
SUC	Standard Urine Culture
UTI	Urinary Tract Infection
VMD	Very Major Discrepancy
VME	Very Major Error

## Appendix A

CLSI References for Workflow Development:

Step I: Choice of disk diffusion as initial standard comparator method: see M52 [22] 1st edition page 3 for the definition of comparator method and page 27, Table 4 for the choice of comparator method; see M100 [19] 35th edition page xii for reclassification of disk diffusion from a “reference” method to a “standard” method.

Step II: Use of broth microdilution as a reference comparator method to resolve discrepancies: see M52 1st edition page 31, Section 3.8.8.

Step III: Use of triplicate testing to resolve discrepancies: see M52 1st edition pages 30–31, Sections 3.8.7–3.8.8.

Step IV: Results interpreted per M52 1st edition page 31, Section 3.8.8 and BIT Part C.
