# Supplementary material for: Full Validation of Pooled Antibiotic Susceptibility Testing Using CLSI Methods and Performance Criteria in UTI Pathogens
Source: Antibiotics (Basel). 2025 Nov 20;14(11):1168. doi: 10.3390/antibiotics14111168 (PMC12649222; doi:10.3390/antibiotics14111168)
Supplement: Supplementary file 1 [file antibiotics-14-01168-s001.zip › antibiotics-3946277-supplementary.pdf]

Supplemental Tables

Supplemental Table S1. Monomicrobial Enterobacterales Numbers of Discrepancies and Errors

Antibiotic	After Step 1: P-AST vs Disk Diffusion			After Step 2: P-AST vs Broth Microdilution			Final (After Step 3) Repeat Broth Microdilution		
	Very Major Discrepancy (VMD)	Major Discrepancy (MD)	Minor Discrepancy (mD)	Very Major Error (VME)	Major Error (ME)	Minor Error (mE)	Very Major Error (VME)	Major Error (ME)	Minor Error (mE)
Amoxicillin/Clavulanate	0	3	11	0	1	2	0	0	1
Ampicillin	0	0	0	0	0	0	0	0	0
Ampicillin/Sulbactam	0	2	9	0	0	5	0	0	4
Cefaclor	0	0	2	0	0	1	0	0	0
Cefazolin	2	1	6	1	0	0	0	0	0
Cefepime	0	0	4	0	0	4	0	0	3
Ceftazidime	0	1	2	0	0	2	0	0	2
Ceftriaxone	0	1	0	0	0	0	0	0	1
Ciprofloxacin	0	1	6	0	0	2	0	0	1
Doxycycline	0	1	2	0	1	0	0	0	1
Fosfomycin	0	2	1	0	1	1	0	0	2
Gentamicin	0	1	2	0	1	2	0	0	1
Levofloxacin	0	2	2	0	0	1	0	0	1
Meropenem	0	0	1	0	0	0	0	0	0
Nitrofurantoin	2	0	8	0	0	4	0	0	3
Piperacillin/Tazobactam	0	0	8	1	0	1	0	0	2
Sulfamethoxazole/Trimethoprim	0	1	0	0	0	0	0	0	0
Trimethoprim	0	1	1	0	1	0	0	0	0

Supplemental Table S2. Monomicrobial Enterococci Numbers of Discrepancies and Errors

Antibiotic	After Step 1: P-AST vs Disk Diffusion			After Step 2: P-AST vs Broth Microdilution			Final (After Step 3) Repeat Broth Microdilution		
	Very Major Discrepancy (VMD)	Major Discrepancy (MD)	Minor Discrepancy (mD)	Very Major Error (VME)	Major Error (ME)	Minor Error (mE)	Very Major Error (VME)	Major Error (ME)	Minor Error (mE)
Ampicillin	0	0	0	0	0	0	0	0	0
Ciprofloxacin	4	0	22	0	0	1	0	0	1
Doxycycline	7	0	23	0	0	5	0	0	3
Fosfomycin	1	0	1	0	0	1	0	0	1
Levofloxacin	1	0	2	0	0	0	0	0	0
Linezolid	7	2	8	0	1	2	0	0	1
Nitrofurantoin	0	0	1	0	0	2	0	0	0
Vancomycin	0	1	4	0	0	1	0	0	0

Supplemental Table S3. Monomicrobial Staphylococci Numbers of Discrepancies and Errors

Antibiotic	After Step 1: P-AST vs Disk Diffusion			After Step 2: P-AST vs Broth Microdilution			Final (After Step 3) Repeat Broth Microdilution		
	Very Major Discrepancy (VMD)	Major Discrepancy (MD)	Minor Discrepancy (mD)	Very Major Error (VME)	Major Error (ME)	Minor Error (mE)	Very Major Error (VME)	Major Error (ME)	Minor Error (mE)
Ciprofloxacin	1	1	4	0	1	1	0	0	1
Doxycycline	1	0	6	0	0	2	0	0	1
Gentamicin	0	0	2	0	0	2	0	0	1
Levofloxacin	2	1	4	0	1	2	0	0	1
Linezolid	2	1	6	0	1	0	0	0	0
Nitrofurantoin	0	0	1	0	0	0	0	0	0
Sulfamethoxazole/Trimethoprim	4	2	0	0	1	0	0	1	0
Trimethoprim	2	1	1	0	0	0	0	0	0
Vancomycin	0	2	1	0	2	1	0	0	0

Supplemental Table S4. Monomicrobial *Pseudomonas aeruginosa* Numbers of Discrepancies and Errors

Antibiotic	After Step 1: P-AST vs Disk Diffusion			After Step 2: P-AST vs Broth Microdilution			Final (After Step 3) Repeat Broth Microdilution		
	Very Major Discrepancy (VMD)	Major Discrepancy (MD)	Minor Discrepancy (mD)	Very Major Error (VME)	Major Error (ME)	Minor Error (mE)	Very Major Error (VME)	Major Error (ME)	Minor Error (mE)
Cefepime	0	1	2	0	1	0	0	0	0
Ceftazidime	1	0	3	0	0	1	0	1	1
Ciprofloxacin	0	0	4	0	0	3	0	0	2
Gentamicin	3	0	6	0	0	0	0	0	0
Levofloxacin	0	0	1	0	0	0	0	0	0
Meropenem	0	1	1	0	1	0	0	0	1
Piperacillin/Tazobactam	1	0	1	0	0	0	0	0	0

Supplemental Table S5. Monomicrobial *Acinetobacter* Species Numbers of Discrepancies and Errors

Antibiotic	After Step 1: P-AST vs Disk Diffusion			After Step 2: P-AST vs Broth Microdilution			Final (After Step 3) Repeat Broth Microdilution		
	Very Major Discrepancy (VMD)	Major Discrepancy (MD)	Minor Discrepancy (mD)	Very Major Error (VME)	Major Error (ME)	Minor Error (mE)	Very Major Error (VME)	Major Error (ME)	Minor Error (mE)
Ampicillin/Sulbactam	0	0	2	0	0	1	0	0	1
Cefepime	2	0	6	1	0	3	0	0	4
Ceftazidime	0	0	1	0	0	0	0	0	0
Ceftriaxone	0	0	4	0	0	2	0	0	2
Ciprofloxacin	0	0	0	0	0	0	0	0	0
Gentamicin	1	0	1	0	0	0	0	0	0
Levofloxacin	0	0	6	1	0	0	0	0	0
Meropenem	1	0	0	1	0	0	0	0	0
Piperacillin/Tazobactam	0	0	1	0	0	1	0	0	1
Sulfamethoxazole/Trimethoprim	2	0	3	0	0	0	0	0	0

Supplemental Table S6. Polymicrobial Enterobacterales Numbers of Discrepancies and Errors

Antibiotic	After Step 1: P-AST vs Disk Diffusion			After Step 2: P-AST vs Broth Microdilution			Final (After Step 3) Repeat Broth Microdilution		
	Very Major Discrepancy (VMD)	Major Discrepancy (MD)	Minor Discrepancy (mD)	Very Major Error (VME)	Major Error (ME)	Minor Error (mE)	Very Major Error (VME)	Major Error (ME)	Minor Error (mE)
Amoxicillin/Clavulanate	0	4	7	0	3	3	0	0	4
Ampicillin	1	2	1	1	2	0	1	0	0
Ampicillin/Sulbactam	3	6	6	2	1	6	0	1	6
Cefaclor	0	0	2	0	0	2	0	0	1
Cefazolin	1	0	1	1	0	0	1	0	0
Cefepime	0	0	3	0	0	2	0	0	1
Ceftazidime	0	0	1	0	0	0	0	0	0
Ceftriaxone	0	2	3	0	0	0	0	0	0
Ciprofloxacin	0	5	14	0	0	8	0	0	4
Doxycycline	0	0	14	0	1	6	0	0	0
Fosfomycin	1	0	6	0	0	4	0	0	2
Gentamicin	4	2	1	0	0	2	0	0	0
Levofloxacin	0	3	12	0	0	8	0	0	3
Meropenem	2	0	3	1	0	2	0	0	1
Nitrofurantoin	0	0	10	0	0	7	0	0	5
Piperacillin/Tazobactam	3	1	12	1	1	2	0	1	2
Sulfamethoxazole/Trimethoprim	0	16	1	0	0	0	0	0	0
Trimethoprim	2	15	4	0	0	0	0	0	0

Supplemental Table S7. Polymicrobial Enterococci Numbers of Discrepancies and Errors

Antibiotic	After Step 1: P-AST vs Disk Diffusion			After Step 2: P-AST vs Broth Microdilution			Final (After Step 3) Repeat Broth Microdilution		
	Very Major Discrepancy (VMD)	Major Discrepancy (MD)	Minor Discrepancy (mD)	Very Major Error (VME)	Major Error (ME)	Minor Error (mE)	Very Major Error (VME)	Major Error (ME)	Minor Error (mE)
Ampicillin	1	2	2	1	2	0	1	0	0
Ciprofloxacin	0	5	17	0	0	9	0	0	4
Doxycycline	0	0	13	0	0	5	0	0	0
Fosfomycin	1	1	4	0	0	2	0	0	0
Levofloxacin	0	3	14	0	0	9	0	0	4
Linezolid	0	1	0	0	0	0	0	0	0
Nitrofurantoin	0	0	9	0	0	6	0	0	4
Vancomycin	0	0	1	0	0	1	0	0	0

Supplemental Table S8. Polymicrobial Staphylococci Numbers of Discrepancies and Errors

Antibiotic	After Step 1: P-AST vs Disk Diffusion			After Step 2: P-AST vs Broth Microdilution			Final (After Step 3) Repeat Broth Microdilution		
	Very Major Discrepancy (VMD)	Major Discrepancy (MD)	Minor Discrepancy (mD)	Very Major Error (VME)	Major Error (ME)	Minor Error (mE)	Very Major Error (VME)	Major Error (ME)	Minor Error (mE)
Ciprofloxacin	0	0	1	0	0	0	0	0	0
Doxycycline	1	0	4	0	0	2	0	0	2
Gentamicin	1	0	0	0	0	1	0	0	1
Levofloxacin	0	0	1	0	0	1	0	0	0
Linezolid	0	1	0	0	0	0	0	0	0
Nitrofurantoin	0	0	1	0	0	0	0	0	0
Sulfamethoxazole/Trimethoprim	1	2	0	0	0	0	0	0	0
Trimethoprim	2	1	1	0	0	0	0	0	0
Vancomycin	0	0	1	0	0	1	0	0	0

Supplemental Table S9. Polymicrobial *Pseudomonas aeruginosa* Numbers of Discrepancies and Errors

Antibiotic	After Step 1: P-AST vs Disk Diffusion			After Step 2: P-AST vs Broth Microdilution			Final (After Step 3) Repeat Broth Microdilution		
	Very Major Discrepancy (VMD)	Major Discrepancy (MD)	Minor Discrepancy (mD)	Very Major Error (VME)	Major Error (ME)	Minor Error (mE)	Very Major Error (VME)	Major Error (ME)	Minor Error (mE)
Cefepime	1	0	0	0	0	0	0	0	0
Ceftazidime	1	0	1	1	0	0	0	0	0
Ciprofloxacin	0	0	2	0	0	0	0	0	0
Gentamicin	4	0	0	0	0	1	0	0	1
Levofloxacin	0	0	0	0	0	0	0	0	0
Meropenem	1	0	6	0	0	4	0	0	2
Piperacillin/Tazobactam	0	0	2	1	0	0	0	0	0

Supplemental Table S10. Polymicrobial *Acinetobacter* Species Numbers of Discrepancies and Errors

Antibiotic	After Step 1: P-AST vs Disk Diffusion			After Step 2: P-AST vs Broth Microdilution			Final (After Step 3) Repeat Broth Microdilution		
	Very Major Discrepancy (VMD)	Major Discrepancy (MD)	Minor Discrepancy (mD)	Very Major Error (VME)	Major Error (ME)	Minor Error (mE)	Very Major Error (VME)	Major Error (ME)	Minor Error (mE)
Ampicillin/Sulbactam	1	0	0	0	0	0	0	0	0
Cefepime	0	0	0	0	0	0	0	0	0
Ceftazidime	0	0	2	0	0	1	0	0	1
Ceftriaxone	1	2	7	0	0	1	0	0	1
Ciprofloxacin	0	0	0	0	0	0	0	0	0
Gentamicin	1	0	0	0	0	1	0	0	1
Levofloxacin	0	0	0	0	0	0	0	0	0
Meropenem	0	0	2	0	0	1	0	0	0
Piperacillin/Tazobactam	0	0	6	0	0	0	0	0	0
Sulfamethoxazole/Trimethoprim	1	0	1	0	0	0	0	0	0

Supplemental Table S11. Precision

Organism Group	Antibiotic	Total n	% Category Equivalency
Enterobacterales	Amoxicillin/Clavulanate	21	95.2
	Ampicillin	21	100
	Ampicillin/Sulbactam	21	95.2
	Cefaclor	21	100
	Cefazolin	21	100
	Cefepime	21	95.2
	Ceftazidime	21	95.2
	Ceftriaxone	21	100
	Ciprofloxacin	21	100
	Doxycycline	21	95.2
	Fosfomycin	21	100
	Gentamicin	21	100
	Levofloxacin	21	100
	Meropenem	21	100
	Nitrofurantoin	21	100
	Piperacillin/Tazobactam	21	95.2
	Sulfamethoxazole/Trimethoprim	21	100
	Trimethoprim	21	100
Enterococci	Ampicillin		100
	Ciprofloxacin	21	95.2
	Doxycycline	21	100
	Fosfomycin	21	100
	Levofloxacin	21	100
	Linezolid	21	100
	Nitrofurantoin	21	100
	Vancomycin	21	100
Staphylococci	Ciprofloxacin	21	100
	Doxycycline	21	95.2
	Gentamicin	21	100
	Levofloxacin	21	100
	Linezolid	21	100
	Nitrofurantoin	21	95.2
	Sulfamethoxazole/Trimethoprim	21	100
	Trimethoprim	21	100
	Vancomycin	21	100

Organism Group	Antibiotic	Total n	% Category Equivalency
Pseudomonas aeruginosa	Cefepime	21	100
	Ceftazidime	21	100
	Ciprofloxacin	21	100
	Gentamicin	21	100
	Levofloxacin	21	100
	Meropenem	21	100
	Piperacillin/Tazobactam	21	100
Acinetobacter species	Ampicillin/Sulbactam	21	100
	Cefepime	21	100
	Ceftazidime	21	100
	Ceftriaxone	21	100
	Ciprofloxacin	21	100
	Gentamicin	21	100
	Levofloxacin	21	100
	Meropenem	21	100
	Piperacillin/Tazobactam	21	100
	Sulfamethoxazole/Trimethoprim	21	100
